# Earliest accumulation of β-amyloid occurs within the default-mode network and concurrently affects brain connectivity

**DOI:** 10.1038/s41467-017-01150-x

**Published:** 2017-10-31

**Authors:** Sebastian Palmqvist, Michael Schöll, Olof Strandberg, Niklas Mattsson, Erik Stomrud, Henrik Zetterberg, Kaj Blennow, Susan Landau, William Jagust, Oskar Hansson

**Affiliations:** 1 Clinical Memory Research Unit, Department of Clinical Sciences, Malmö, Lund University, Lund, Sweden; 2 Department of Neurology, Skåne University Hospital, Lund, Sweden; 3 Wallenberg Centre for Molecular and Translational Medicine and the Department of Psychiatry and Neurochemistry, University of Gothenburg, Gothenburg, Sweden; 4 Memory Clinic, Skåne University Hospital, Malmö, Sweden; 5 Department of Psychiatry and Neurochemstry, Institute of Neuroscience and Physiology, the Sahlgrenska Academy at the University of Gothenburg, Mölndal, Sweden; 6 Clinical Neurochemistry Laboratory, Sahlgrenska University Hospital, Mölndal, Sweden; 7 Department of Molecular Neuroscience, UCL Institute of Neurology, Queen Square, London UK; 8 Helen Wills Neuroscience Institute, University of California, Berkeley, USA

## Abstract

It is not known exactly where amyloid-β (Aβ) fibrils begin to accumulate in individuals with Alzheimer’s disease (AD). Recently, we showed that abnormal levels of Aβ42 in cerebrospinal fluid (CSF) can be detected before abnormal amyloid can be detected using PET in individuals with preclinical AD. Using these approaches, here we identify the earliest preclinical AD stage in subjects from the ADNI and BioFINDER cohorts. We show that Aβ accumulation preferentially starts in the precuneus, medial orbitofrontal, and posterior cingulate cortices, i.e., several of the core regions of the default mode network (DMN). This early pattern of Aβ accumulation is already evident in individuals with normal Aβ42 in the CSF and normal amyloid PET who subsequently convert to having abnormal CSF Aβ42. The earliest Aβ accumulation is further associated with hypoconnectivity within the DMN and between the DMN and the frontoparietal network, but not with brain atrophy or glucose hypometabolism. Our results suggest that Aβ fibrils start to accumulate predominantly within certain parts of the DMN in preclinical AD and already then affect brain connectivity.

## Abstract (web summary)

Abnormal levels of Aβ42 in the cerebrospinal fluid occur prior to a positive amyloid PET scan in the brain of individuals with Alzheimer’s disease and here the authors use this temporal pattern to identify individuals with very early stage AD. They show that Aβ fibrils start to accumulate in some of the regions of the default mode network and affect brain connectivity before neurodegeneration occurs.

## Introduction

Accumulation of aggregated amyloid-β (Aβ) in the brain is believed to be the first pathological mechanism of Alzheimer’s disease (AD)^1^. Previous studies have shown that cerebral deposition of Aβ fibrils can occur for decades before an individual with AD reaches the dementia stage^2^. The widespread cerebral distribution of Aβ in later AD stages is well-established^3, 4^, however it has been difficult to accurately identify the initial brain regions of Aβ accumulation due to the long time-lag between the start of the pathophysiology and onset of symptoms.

A neuropathological staging of different Aβ phases in AD has been proposed based on post-mortem examinations and it has been shown that the first phase is characterized by Aβ deposits throughout the frontal, parietal, temporal, and occipital neocortex^5^. Studies employing positron emission tomography (PET) to visualize fibrillar Aβ deposits have also attempted to identify early Aβ-accumulating regions. For example, when analyzing the earliest data from AD mutation-carriers, most cortical areas except for the sensorimotor cortex showed Aβ accumulation as early as 15 years before the expected onset of symptoms^6^. A shortcoming of such studies is the cross-sectional design, which precludes conclusions about longitudinal events. Another issue is that it has previously been difficult to identify the earliest preclinical AD cases; when mixing such cases with later asymptomatic AD cases that have been accumulating Aβ fibrils for years, the cortical sites of accumulation of Aβ identified are widespread.

In the present study, we applied a novel methodology of combining data obtained regarding Aβ levels in the brain via PET, and levels of the Aβ42 peptide in cerebrospinal fluid (CSF), to identify individuals who have just recently started to accumulate Aβ in the brain. Previous cross-sectional studies indicate that Aβ42 levels decrease in the CSF as Aβ starts to accumulate, before fibrillar plaques can be detected with PET^7–11^. Recently, this was confirmed longitudinally in a cohort of more than 400 non-demented individuals where we found that those with abnormally low CSF levels of Aβ42, but with still normal Aβ levels detected by PET (CSF+/PET−), accumulated Aβ fibrils longitudinally at a similar rate as those who had both abnormal CSF levels of Aβ42 and abnormal levels of Aβ detected by PET (CSF+/PET+) and four times higher than those with normal biomarker results on both parameters (CSF−/PET−)^12^. This indicates that individuals with AD may first be defined as being CSF+/PET−, and then progress to CSF+/PET+. It might therefore be possible to identify the earliest stages of preclinical AD by studying individuals with abnormal CSF Aβ42 and normal Aβ PET (CSF+/PET−, also referred to as early Aβ accumulators to indicate their stage in the accumulation of Aβ).

The main hypothesis in the present study was that Aβ fibrils are more prone to start aggregating in certain regions of the brain before they can be found throughout the neocortex, and before neurodegeneration is present. Identifying these regions and examining their properties can provide important information about why Aβ aggregation starts in sporadic AD, which is a crucial step for developing disease-modifying drugs. The aim of the study was therefore to identify such regions by comparing the longitudinal fibrillar Aβ deposition rates in different brain regions of early Aβ accumulators (termed CSF+/PET−) with non-accumulators (termed CSF−/PET−). To avoid bias from non-optimal cut-offs for CSF Aβ42 levels, we also examined the correlation between continuous CSF Aβ42 levels and the regional Aβ PET signal in PET negative subjects. We performed the analyses in 68 predefined anatomical regions as well as employing voxelwise statistics. The analyses were performed both in a main cohort and an independent validation cohort. As the main cohort, we used the North American Alzheimer’s Disease Neuroimaging Initiative (ADNI) and included all non-demented participants with CSF Aβ42 and longitudinal Aβ PET data (473 individuals). The Swedish BioFINDER study was used as the validation cohort, from which we included all non-demented participants with baseline CSF Aβ42 and Aβ PET data (406 individuals).

We also examined early regional Aβ accumulation in the presumably earliest Aβ accumulators; those with normal CSF Aβ42 and normal Aβ PET at baseline who converted to abnormal CSF Aβ42 levels at follow-up but still had a normal Aβ levels identified by PET scan (CSF−/PET to CSF+/PET−). Consistently, using different cohorts and methods, we identified a specific set of early Aβ-accumulating regions, which largely corresponded to a functional brain network, the default mode network (DMN). The relationship between early stage Aβ accumulation and whole-brain functional connectivity was examined in detail and revealed that hypoconnectivity, especially within the DMN and between DMN and the frontoparietal network, was associated with low CSF Aβ42 levels in individuals that still had normal Aβ PET scans.

To test the hypothesis that early Aβ accumulation is an upstream mechanism that precedes overtly decreased energy metabolism and cell death, we compared the longitudinal changes in ^18^F-fluorodeoxyglucose (FDG) PET and volumetric magnetic resonance imaging (MRI) in the different groups. Finally, we examined the validity of using CSF/PET groups to stratify Aβ stages by comparing early (CSF+/PET−) and late (CSF+/PET+) accumulators to identify known late regions of Aβ accumulation, including the sensorimotor cortex.

## Results

### Experimental outline and baseline characteristics

We first analyzed the longitudinal results from the ADNI cohort, then replicated the result in the BioFINDER cohort and finally analyzed functional connectivity in BioFINDER.

Baseline characteristics of the ADNI sample and the different CSF/PET groups are provided in Table 1. Early stage Aβ accumulators (CSF+/PET−) had significantly higher prevalence of the *APOE ε4* genotype and a slightly higher neocortical florbetapir standardized uptake value ratio (SUVR), but similar baseline cognition, hippocampal volume, and CSF tau levels as non-accumulators (CSF−/PET−) (Table 1). By contrast, the non-demented late stage Aβ accumulators (CSF+/PET+) showed, at baseline, signs of neurodegeneration with hippocampal atrophy, higher CSF tau levels and worse cognition, and had per definition significantly increased florbetapir SUVRs (Table 1).

**Table 1:** Baseline characteristics of the ADNI cohort

		**A**	**B**	**C**	***p***-**value**
	**Entire ADNI sample**	**CSF−/PET−**	**CSF+/PET−**	**CSF+/PET+**	
*N*	473	218	59	191	
Baseline CN/MCI	37%/63%	47%/53%	44%/56%	23%/77%	A-B = 0.58
					**B**-**C** < **0.001**
					**A**-**C** < **0.001**
Age (years)	72.0 (7.0)	71.0 (7.0)	71.2 (8.1)	73.2 (6.3)	A-B = 0.99
					B-C = 0.20
					**A**-**C** < **0.001**
Gender (women)	47%	47%	46%	48%	0.95
Education (years)	16.5 (2.6)	16.7 (2.6)	17.0 (2.2)	16.1 (2.6)	A-B = 0.47
					**B**-**C** = **0.007**
					**A**-**C** = **0.03**
Presence of the *APOE ε4* allele	40%	16%	46%	65%	**A**-**B** < **0.001**
					**B**-**C** = **0.004**
					**A**-**C** < **0.001**
MMSE (0–30 points)	28.4 (1.7)	28.8 (1.4)	28.8 (1.3)	27.9 (1.8)	A-B = 0.95
					**B**-**C** = **0.001**
					**A**-**C** < **0.001**
Baseline neocortical composite florbetapir SUVR^1^	0.90 (0.21)	0.74 (0.05)	0.78 (0.06)	0.93 (0.09)	**A**-**B** < **0.001**
					**B**-**C** < **0.001**
					**A**-**C** < **0.001**
Years between florbetapir PET scans (range)	2.0 (0.9–4.1)	2.0 (0.9–4.1)	2.0 (1.9–4.1)	2.0 (1.7–3.0)	1.0
Years between FDG PET scans (range)	2.0 (0.8–3.1)	2.0 (0.8–2.7)	2.1 (1.9–3.1)	2.0 (1.8–3.0)	0.72
Years between MRI scans (range)	2.0 (0.4–4.1)	2.0 (0.4–4.1)	2.0 (0.5–4.1)	2.0 (0.6–3.1)	0.92
Hippocampus volume (cm^3^)	7.2 (1.1)	7.5 (1.0)	7.5 (0.9)	6.9 (1.3)	A-B = 0.92
					**B**-**C** < **0.001**
					**A**-**C** < **0.001**
CSF Aβ42 (ng/L)	186 (52)	234 (27)	165 (23)	136 (22)	**A**-**B** < **0.001**
					**B**-**C** < **0.001**
					**A**-**C** < **0.001**
CSF T-tau (ng/L)	79 (46)	59 (26)	60 (31)	109 (53)	A-B = 0.95
					**B**-**C** < **0.001**
					**A**-**C** < **0.001**
CSF P-tau (ng/L)	39 (23)	28 (12)	30 (26)	54 (25)	A-B = 0.19
					**B**-**C** < **0.001**
					**A**-**C** < **0.001**

CN cognitively normal, CSF cerebrospinal fluid, CSF+*/−* abnormal/normal CSF Aβ42 levels, MCI mild cognitive impairment, MMSE Mini Mental State Examination, *N* number of subjects, PET+*/−* abnormal/normal florbetapir SUVR, SD standard deviation, SUVR standardized uptake value ratio (using a composite reference region) corrected for partial volume errors. Comparison between non-accumulators (CSF−/PET−), early Aβ accumulators (CSF+/PET−), and late Aβ accumulators (CSF+/PET+). Values are given in mean (SD) if not otherwise specified. Groups were compared with Mann–Whitney *U* statistics if significant after the Kruskal–Wallis test. Significant *p* values are in bold. The CSF−/PET+group was not included in this table as a separate group since they were not used in any analysis and they were also too few to be used in the group comparisons (*n* = 5)

### Identifying early Aβ-accumulating regions

**Fig. 1:**
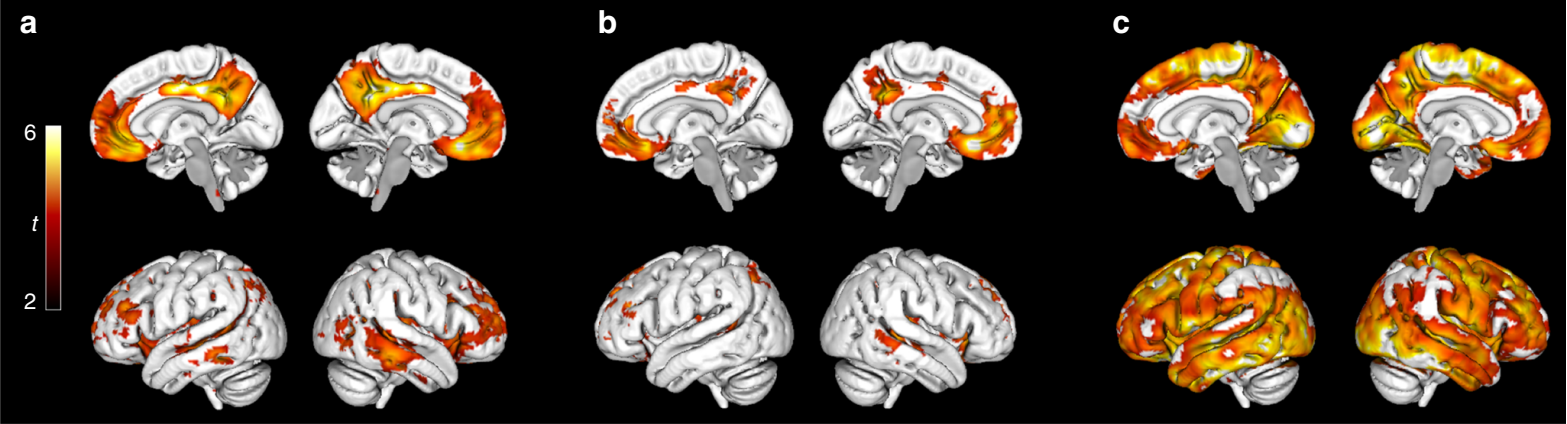
Regions of Aβ accumulation from longitudinal voxelwise analyses in ADNI. **a** shows the regions where Aβ fibrils start to accumulate by comparing the annual florbetapir SUVR rate during 2 years between early stage Aβ accumulators (CSF+/PET−, *n* = 59) and non-accumulators (CSF−/PET−, *n* = 218). The lateral and medial projections in **a** show that the most significantly increased accumulation rate among the early Aβ accumulators was located in the posterior cingulate cortex, the precuneus, and the medial orbitofrontal cortex. A PET-data-derived ROI of these early-accumulating Aβ regions is available at http://biofinder.se **b** confirms the regions in **a** without biases from a specific CSF Aβ42 cut-off. Here, we performed voxelwise correlations between annual florbetapir SUVR rates and CSF Aβ42 levels in Aβ PET negative individuals (*n* = 277). To contrast the early stage Aβ regions, **c** shows the regions with significantly increased annual SUVR rate in late stage Aβ accumulators (CSF+/PET+, *n* = 191) compared with non-accumulators (CSF−/PET−). A widespread pattern of Aβ accumulation is seen in these non-demented CSF+/PET+ subjects. Voxelwise two-sample *t*-tests were used and all analyses in **a**–**c** are adjusted for age and gender. The significant threshold was set at *p* < 0.001. The red and yellow colors illustrate significant *t* values according to the scale on the left

To identify early Aβ accumulation regions, longitudinal voxelwise comparisons of accumulation rates (SUVR/year) were performed between CSF+/PET− (early Aβ accumulators) and CSF−/PET− (non-accumulators). The results are shown in Fig. 1a (see Supplementary Fig. 1 for whole-brain axial images). The brain regions showing significant increases in Aβ PET signal over time in CSF+/PET− cases included the medial orbitofrontal cortex, anterior cingulate cortex, posterior, and isthmus cingulate cortex, precuneus and to a lesser extent temporal regions. Similar significant regions were identified by correlating CSF Aβ42 levels with the accumulation rates in PET− subjects to remove potential biases from the Aβ42 cut-off (Fig. 1b). In the region of interest (ROI)-based comparisons, 15 of 68 cortical regions had an increased rate in the CSF+/PET− subjects compared to the CSF−/PET− subjects (Table 2 and Supplementary Movie 1). The significant differences were in agreement with the voxelwise analyses and were mainly seen in the orbitofrontal cortex, precuneus, insula, and the posterior/isthmus cingulate cortex. On average the yearly Aβ accumulation rate of the CSF+/PET− group was ~4–5 times that of the CSF−/PET− group in the significant brain regions (Table 2). All results were corrected for multiple comparisons and adjusted for gender, age, and time between PET scans. To account for atrophy as a confounding factor, we also adjusted all significant models for the annual change in cortical thickness of each ROI. After this adjustment, all 15 regions still differed significantly between the two groups. The 15 early Aβ regions were also significant when adjusting for diagnosis (MCI or healthy control). Finally, we adjusted for *APOE* genotype to examine if different early Aβ patterns were observed depending on whether one carried an *APOE* ε4 allele, but the same regions as in Table 2 were found (details shown in Supplementary Table 1). The distinct pattern of early Aβ accumulation (Fig. 1a, b, Table 2) can be contrasted to the more global Aβ accumulation pattern seen in late, but still non-demented, Aβ accumulators (CSF+/PET+) compared with non-accumulators (CSF−/PET−) (Fig. 1c).

**Table 2:** Annual Aβ accumulation rates in the significant early Aβ regions in ADNI

Region	CSF+/PET−	CSF−/PET−	*p*-value
Posterior cingulate cortex, right	2.7% (1.8–3.6)	0.9% (0.5–1.3)	0.000051
Medial orbitofrontal cortex, left	2.5% (1.4–3.6)	0.4% (−0.2–1.0)	0.00026
Medial orbitofrontal cortex, right	2.5% (1.3–3.7)	0.4% (−0.1–1.0)	0.00038
Precuneus, left	2.3% (1.4–3.3)	0.7% (0.2–1.2)	0.00051
Rostral anterior cingulate cortex, right	2.0% (1.3–2.8)	0.5% (0.0–1.0)	0.00081
Lateral orbitofrontal cortex, left	1.8% (0.9–2.7)	0.3% (−0.1–0.7)	0.0011
Insula, right	1.8% (0.8–2.8)	0.2% (−0.3–0.7)	0.0025
Isthmus cingulate cortex, left	2.0% (1.1–2.8)	0.5% (0.0–1.0)	0.0027
Rostral anterior cingulate cortex, left	1.9% (1.1–2.6)	0.6% (0.1–1.1)	0.0031
Isthmus cingulate cortex, right	2.1% (1.2–2.9)	0.5% (0.0–1.0)	0.0032
Precuneus, right	1.9% (1.1–2.8)	0.6% (0.2–1.1)	0.0041
Superior frontal cortex, left	1.2% (0.5–2.0)	0.2% (−0.2–0.5)	0.0053
Transverse temporal gyrus, right	0.8% (−0.3–1.9)	−0.8% (−1.5–−0.2)	0.0074
Insula, left	1.5% (0.7–2.3)	0.2% (−0.3–0.7)	0.0085
Posterior cingulate cortex, left	2.1% (1.2–3.0)	0.9% (0.5–1.4)	0.01

CSF+ cerebrospinal fluid Aβ < 192 ng/L, PET+ >0.87 SUVR, ROI region of interest, SUVR standardized uptake value ratio (using a composite reference region). Comparisons in ADNI between early Aβ accumulators (CSF+/PET−) and non-accumulators (CSF−/PET−) subjects using general linear models with the Aβ PET SUVR change/year in each of the 68 cortical Freesurfer ROI:s as the dependent variable and CSF/PET groups, sex, age, and time between PET scans as covariates. Data are given in mean values (95% CI of the mean) in order of significance and Aβ accumulation is shown in % yearly SUVR change. Only the 15 significant regions after the Benjamini & Hochberg correction are shown. These regions were also significant when adjusting for changes in cortical thickness over 2 years (in each region) and diagnosis (MCI or healthy control)

### Early Aβ accumulating regions in CSF converters

Previous studies have found that non-demented CSF−/PET− individuals, that during follow-up show abnormal CSF Aβ42 levels, might represent the earliest preclinical AD phase^13, 14^. We therefore examined CSF−/PET− subjects who, after 2 years, converted to CSF+/PET− (referred to as CSF converters). In the ADNI cohort there were 112 CSF−/PET− subjects with longitudinal CSF data that could be included in this analysis. At follow-up investigation, 11 of these 112 subjects had converted to abnormal CSF Aβ42 levels (CSF+/PET−) while 101 subjects remained stable with normal CSF Aβ42 levels (CSF−/PET−). Significant differences in Aβ accumulation rate (Aβ PET SUVR/year) between the two groups were seen in the left posterior cingulate and right medial orbitofrontal cortex (*p* = 0.02–0.03). The group comparisons were still significant after adjustment of age, gender, and time between PET scans, but probably because of the limited statistical power (*n* = 11) there were no significant differences after multiple comparison correction. Given the agreement with the previously identified regions (Fig. 1a, b and Table 2), we interpreted the identified regions as true and not a statistical type 1 error. For the CSF converters, the SUVR increased significantly with mean rates of +2.3–2.5% per year in the left posterior cingulate cortex and the right medial orbitofrontal cortex. No significant increase was seen in these regions among the non-converters and the corresponding mean rates were +0.3% (95% CI −0.24 to +0.90%) and +0.3% (95% CI −0.7 to +1.2%).

### Aβ accumulation and functional brain networks

**Fig. 2:**
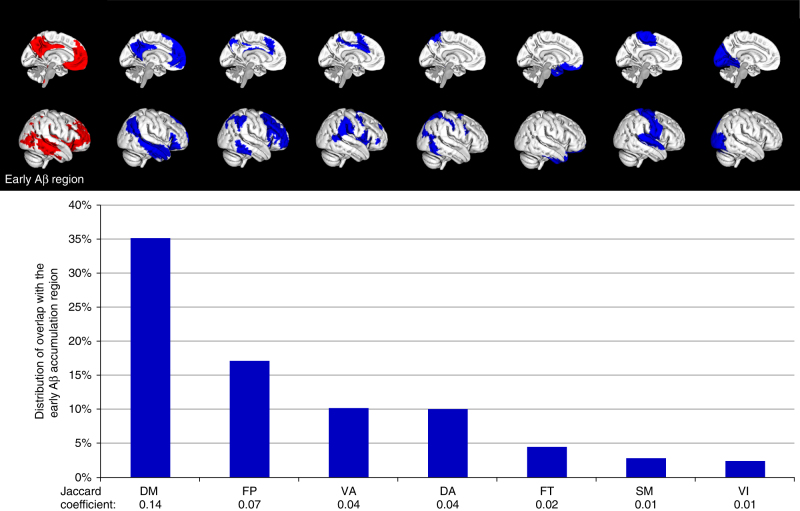
Distribution of early Aβ accumulation among functional networks in ADNI. The bars show the distribution of the overlap between the significant cluster of early Aβ accumulation (shown in red, see also Fig. 1a) and the functional network ROIs (shown in blue above the bars). The sum of the seven bars is therefore 100%. The pattern of early stage Aβ fibrils overlapped mostly with the DM followed by the FP network. The Jaccard coefficient describes the similarity between the early Aβ accumulation region and the functional network ROIs. It is calculated as the overlap between the early Aβ region and the network ROI/(early Aβ region + network ROI - overlap between early Aβ region and the network ROI). DA dorsal attention, DM default mode, FP frontoparietal, FT frontotemporal, SM sensory motor, VA ventral attention, VI visual network

The regional distribution of the early accumulation of cortical Aβ fibrils (Fig. 1a, b) resembled the brain regions involved in the DMN. This led us to examine early accumulation of Aβ fibrils within the different functional brain networks. We applied the early accumulation regions from Fig. 1a to a functional connectivity network atlas^15^ in order to determine the involvement of the different networks in early Aβ accumulation. Figure 2 shows that early Aβ accumulation predominantly overlapped with the DMN (Jaccard coefficient, JC, 0.14), but also with the frontoparietal network (JC 0.07). The networks that were the least involved in early Aβ accumulation were the sensorimotor (JC 0.01) and visual networks (JC 0.01).

### Validation of our Aβ staging by identifying late Aβ regions

**Fig. 3:**
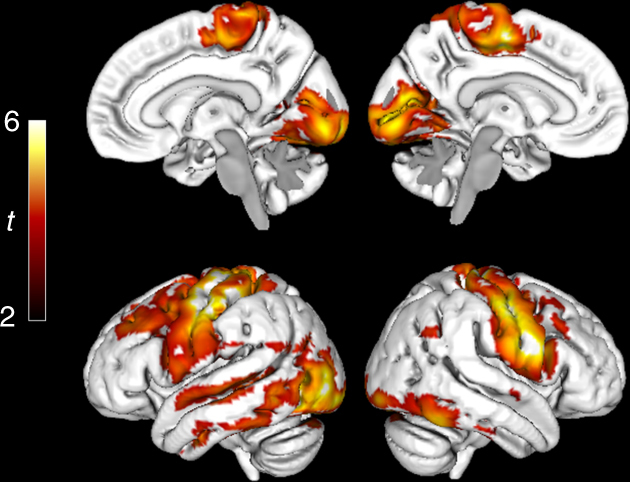
Late Aβ accumulation regions in ADNI. Voxelwise comparisons of annual SUVR change in early Aβ accumulators (CSF+/PET−, *n* = 59) and late Aβ accumulators (CSF+/PET+, *n* = 191). A significantly increased SUVR rate is seen in the sensorimotor cortex, occipital lobe, and dorsal temporal lobe in late compared with early Aβ accumulators. Voxelwise two-sample *t*-tests were used and all comparisons were adjusted for age and gender. The significance threshold was set at *p* < 0.001. The red and yellow colors illustrate significant *t* values according to the scale on the left

We have used the CSF+/PET− subjects as a proxy for early stage Aβ accumulators and compared them with CSF−/PET− subjects (non-accumulators) to identify early Aβ regions. If our CSF/PET groups are valid for staging Aβ phases, we should also be able to compare early Aβ accumulators and CSF+/PET+ subjects (our proxy for late stage Aβ accumulators) to identify known regions of late Aβ accumulation. The results from the ROI-based comparisons are shown in Table 3 and Supplementary Movie 2. The largest differences were seen in the precentral, postcentral, paracentral, pericalcarine, and lingual regions. This means that our proxy group for late Aβ accumulation (CSF+/PET+) had a higher accumulation rate of the Aβ PET signal over time compared with early Aβ accumulators (CSF+/PET−) around the sensorimotor cortex and occipital lobe. Note that these significant, late Aβ regions did not overlap with the early Aβ regions (compare Tables 2 and 3). The corresponding voxelwise comparisons between early and late accumulators are shown in Fig. 3 and highlight similar late regions (greatest significant difference in the sensorimotor cortex and parts of the occipital lobe).

**Table 3:** Annual Aβ accumulation rates in the significant late Aβ regions in ADNI

Region	CSF+/PET−	CSF+/PET+	*p*-value
Precentral, right	0.1% (−0.5–0.7)	2.1% (1.7–2.6)	0.000002
Postcentral, right	0.1% (−0.7–0.9)	2.3% (1.8–2.7)	0.000005
Pericalcarine, left	0.2% (−0.8–1.3)	2.8% (2.2–3.6)	0.000006
Postcentral, left	0.2% (−0.5–1.2)	2.4% (1.9–2.8)	0.000008
Precentral, left	0.3% (−0.3–1.0)	2.3% (1.9–2.9)	0.000024
Paracentral, left	0.6% (−0.2–1.4)	2.4% (1.9–2.8)	0.000057
Lingual, left	0.5% (−0.7–1.9)	2.8% (2.2–3.4)	0.00019
Lateral occipital, left	0.4% (−0.4–1.3)	2.3% (1.8–2.9)	0.00026
Pericalcarine, right	0.4% (−0.6–1.5)	2.9% (2.3–3.6)	0.0003
Paracentral, right	0.6% (−0.2–2.1)	2.2% (1.7–2.7)	0.00038
Fusiform, left	1.1% (0.3–2.0)	2.4% (2.0–2.8)	0.00049
Lingual, right	0.6% (−0.4–1.8)	2.8% (2.2–3.5)	0.0006
Cuneus, left	−0.3% (−1.2–0.8))	2.0% (1.4–2.8)	0.00062
Middle temporal, left	1.1% (0.3–2.0)	2.2% (1.9–2.6)	0.0007
Lateral occipital, right	0.6% (−0.1–1.4)	2.4% (1.9–3.1)	0.00088
Fusiform, right	0.9% (0.0–1.8)	2.4% (1.9–2.9)	0.0017
Inferior temporal, right	1.3% (0.5–2.2)	2.4% (1.9–2.8)	0.0022
Inferior temporal, left	1.5% (0.7–2.5)	2.6% (2.2–3.1)	0.003
Superior temporal sulcus, left	1.4% (0.6–2.3)	2.4% (1.9–2.8)	0.0047
Caudal middle frontal, left	1.3% (0.5–2.1)	2.5% (2.0–3.1)	0.0054
Superior parietal, left	1.0% (0.2–1.9)	1.9% (1.5–2.3)	0.0074
Superior frontal, left	1.2% (0.5–2.0)	1.9% (1.5–2.3)	0.013

CSF+ cerebrospinal fluid Aβ42 < 192 ng/L, PET+ >0.872 SUVR, ROI region of interest, SUVR standardized uptake value ratio (using a composite reference region). Comparisons in ADNI between early Aβ accumulators (CSF+/PET−) and late Aβ accumulators (CSF+/PET+) to identify late stage Aβ accumulation regions. The analysis was performed using general linear models with the Aβ PET SUVR change/year in each ROI as the dependent variable and CSF/PET groups, sex, age, and time between PET scans as covariates. Data are given in mean values (95% CI of the mean) in order of significance and Aβ accumulation is shown in % yearly SUVR change. Only significant regions after the Benjamini & Hochberg correction are shown

### Atrophy and glucose metabolism in relation to Aβ accumulation

**Fig. 4:**
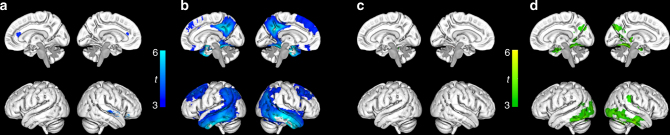
Group comparisons of annual change in brain volume and glucose metabolism in ADNI. **a** Voxel-based morphometry (VBM) comparison of annual MRI change over 2 years in early Aβ accumulators (CSF+/PET−, *n* = 59) compared with non-accumulators (CSF−/PET−, *n* = 218). The results are adjusted for age, gender, and intracranial volume. The contrast is reduction in cortical thickness in early compared with non-accumulators and shows no clear pattern of longitudinal atrophy in the early Aβ accumulators (CSF+/PET−). **b** The same VBM analysis as in **a**, but with a comparison between non-accumulators and late Aβ accumulators (CSF+/PET+, *n* = 191). The contrast is reduction in late compared with non-accumulators and shows a typical AD atrophy pattern in the non-demented late Aβ accumulators (CSF+/PET+). **c** Voxelwise analysis of annual FDG PET change over 2 years in non-accumulators (*n* = 153) compared with early Aβ accumulators (*n* = 41). No significant difference is seen. **d** The same FDG PET analysis as above, but with a comparison between non-accumulators and late Aβ accumulators (*n* = 124). The contrast is reduction in glucose metabolism in late compared with early Aβ accumulators and shows a temporal and to a lesser extent parietal reduction in metabolism in the non-demented late Aβ accumulators. Voxelwise two-sample *t*-tests were used and all results in **a**–**d** were adjusted for age and gender. The VBM analysis was also adjusted for total intracranial volume. Only voxels with a *p* < 0.001 are shown. The colors illustrate significant *t* values according to the scales

To test the hypothesis that atrophy, as a marker of neuronal and synaptic degeneration, starts after the initiation of Aβ accumulation, we compared the longitudinal change in gray matter (GM) volumes between the CSF/PET groups using voxel-based morphometry (VBM) analysis (Fig. 4a, b and Supplementary Fig. 2A). No clear atrophy pattern was seen in early Aβ accumulators (CSF+/PET−) compared with non-accumulators (CSF−/PET−) (Fig. 4a). In contrast, late Aβ accumulators (CSF+/PET+) exhibited the expected longitudinal atrophy pattern of over time involving mainly temporoparietal regions when comparing them to non-accumulators (Fig. 4b) and early Aβ accumulators (Supplementary Fig. 2A). Similar analyses were made with longitudinal FDG PET data to examine changes in glucose metabolism in relation to early Aβ accumulation. There were no differences in FDG uptake over time between non-accumulators (CSF−/PET−) and early Aβ accumulators (CSF+/PET−) (Fig. 4c), while a cortical metabolic reduction in the temporal lobe was seen in late Aβ accumulators (CSF+/PET+) compared with non-accumulators (CSF−/PET−) (Fig. 4d) and early Aβ accumulators (CSF+/PET−) (Supplementary Fig. 2B).

### Replication of the early Aβ regions in the BioFINDER cohort

**Fig. 5:**
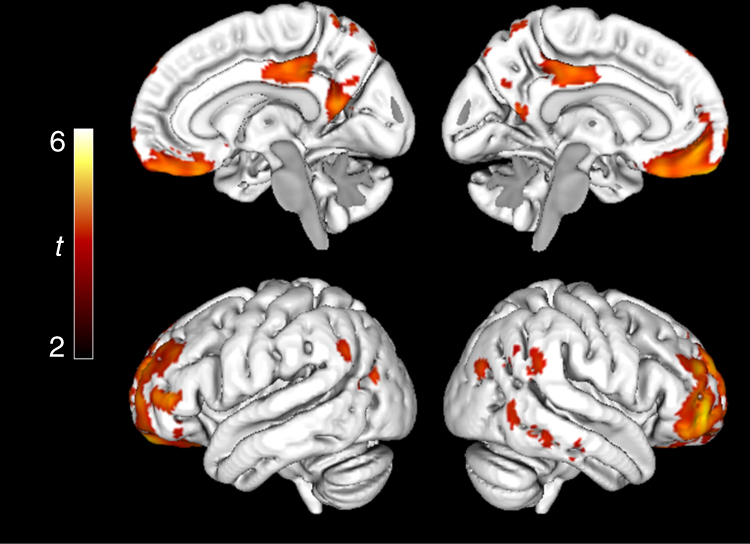
Replication of the early Aβ regions in BioFINDER. Comparison of ^18^F-flutemetamol SUVR between early accumulators (CSF+/PET−, *n* = 30) and non-accumulators (CSF−/PET−, *n* = 219) in BioFINDER to identify early Aβ regions. The highest significance was seen around the posterior regions of the cingulate cortex and the orbitofrontal cortex similar to the results in ADNI (Fig. 1a, b). Note that the BioFINDER analysis included cross-sectional data and fewer subjects compared to ADNI, which results in less statistical power. Voxelwise two-sample *t*-test was used. The significance threshold was set at *p* < 0.001 and the comparison was adjusted for age and gender. The red/yellow colors illustrate significant *t* values according to the scale on the left

To test the generalizability of our results we replicated the early Aβ accumulation regions in the BioFINDER cohort. Only cross-sectional data was available from the BioFINDER study. Although longitudinal comparisons are preferred given our hypothesis, regional differences in Aβ deposition should still be noticeable when comparing CSF+/PET− and CSF−/PET− subjects cross-sectionally. A strength of this cohort is that another Aβ PET ligand (^18^F -flutemetamol) as well as another immunoassay for CSF Aβ (INNOTEST) were used, and the results would therefore be more generalizable if they were replicable in this cohort. The characteristics of BioFINDER is provided in Supplementary Table 2. Similar early Aβ accumulation regions were identified as in ADNI when comparing cross-sectional ^18^F-flutemetamol SUVRs between CSF+/PET− and CSF−/PET− subjects using ROI-based analyses in BioFINDER (Supplementary Table 3). The most significant regions were found in the orbitofrontal cortex, the posterior cingulate cortex, and the precuneus (Supplementary Table 3, which can be compared with ADNI data in Table 2). Further, the cross-sectional voxelwise comparisons between CSF+/PET− and CSF−/PET− subjects in BioFINDER showed significant changes predominantly in the orbitofrontal cortex and posterior cingulate similar to the longitudinal analyses in ADNI (Fig. 5, compare with ADNI data in Fig. 1a, b).

### Early Aβ accumulation and functional connectivity

**Fig. 6:**
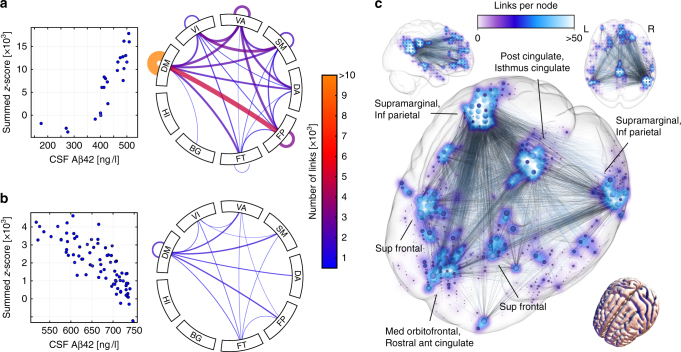
Association between functional connectivity and early Aβ accumulation in BioFINDER. **a** and **b** show CSF Aβ42 correlations with whole-brain resting-state fMRI connectivity in non-demented subjects with normal Aβ PET and QC-passed fMRI data (*n* = 103). Nodes were grouped into the 7 networks and 2 subcortical regions in the connectograms (**a** and **b**). The connectograms show that the significant network component associated with CSF Aβ42 was qualitatively similar for the CSF+/PET− (**a**) and CSF-_low_/PET− (**b**) groups and consisted mostly of intra- and inter-DM connections. Intra-DM dominated and the strongest inter-DM connection was with the frontoparietal network. The network component of the CSF+/PET− group exhibited reduced connectivity with decreasing CSF Aβ42 (**a**), *r* = 0.91, *p* < 0.001. By contrast, in the qualitatively similar network component of the CSF-_low_/PET− group, decreasing Aβ42 was instead associated with increased connectivity (**b**), *r* = −0.85, *p* < 0.001. Panel **c** shows the dominating intra DM-reduction in connectivity for the CSF+/PET− group (**a**). In summary, **a** show that Aβ accumulation measured with CSF Aβ42 in early Aβ accumulators is associated with hypoconnectivity in intra- and inter-DM network links. **b** instead shows that in a population including subjects with even earlier indications of Aβ accumulation (CSF-_low_/PET−) Aβ accumulation is instead associated with hyperconnectivity in similar neuronal connections. Correlation coefficients (*r*) refer to Spearman correlation between summed connectivity and CSF Aβ42 levels. Age, gender, and *APOE* ε4 status was controlled for by partial correlation. Network components correlating with CSF Aβ42 were calculated using a method similar to the NBS algorithm (see “Methods for the BioFINDER study” for statistical details). Acronyms: BG basal ganglia, CSF-_low_ normal CSF Aβ42 levels close to the abnormal cut-off (517–750 ng/L), DA dorsal attention, DM default mode, FP frontoparietal, FT frontotemporal, HI hippocampus, QC quality control, SM sensorimotor, VA ventral attention, VI visual network

Resting-state functional MRI was available in the BioFINDER study and this cohort was therefore used for this analysis. To examine the association between early Aβ accumulation and functional networks beyond the anatomical similarities (Fig. 2) we correlated levels of CSF Aβ42 with whole-brain connectivity (Fig. 6). The groups of interest were the early Aβ accumulators (CSF+/PET−) and biomarker normal individuals with close to abnormal CSF Aβ42 (CSF-_low_/PET−). In the CSF+/PET− group we found a significant network component where reduced connectivity was associated with decreasing levels of CSF Aβ42. The component predominantly consisted of intra-DMN links, but also links between the DMN and the frontoparietal network (Fig. 6a). The summed connectivity on the significant correlation component changed most drastically when CSF Aβ42 levels were closer to the cut-off (400–516 ng/L for the INNOTEST ELISA) and seemed to reach a floor effect as Aβ42 reached levels of around 350 ng/L. Figure 6c depicts the intra-DMN links, which dominate the significant hypoconnectivity component correlating with lower CSF Aβ42 values in the early Aβ accumulators. Furthermore, a significant network component involving the DMN was associated with CSF Aβ42 in the CSF-_low_/PET− group, but with fewer constituent links (Fig. 6b). Interestingly, the correlation was in the opposite direction showing an increased connectivity as CSF Aβ42 levels dropped from normal levels towards the abnormal cut-off. In summary, Fig. 6 indicates that when CSF Aβ42 levels drop within the near abnormal range they might be associated with an increased connectivity in connections involving the DMN. Then, when CSF Aβ42 levels become abnormal and decrease further as Aβ accumulates, they are instead clearly associated with a decreased connectivity within the DMN and mainly between the DMN and the frontoparietal network. The associations with hypoconnectivity and hyperconnectivity were similar when adjusting for CSF P-tau and T-tau, indicating the independent effect of Aβ on the connectivity (Supplementary Fig. 3).

## Discussion

This study examined where Aβ fibrils are more prone to start accumulating, by comparing the longitudinal outcome of non-demented subjects with early signs of Aβ accumulation (CSF+/PET−) to those with no measurable signs of Aβ accumulation (CSF−/PET−), in a large cohort of 473 subjects. The results showed significantly increased rates of Aβ fibril accumulation predominantly in the precuneus, posterior cingulate cortex, and orbitofrontal cortex in this early Aβ stage. When examining subjects with even earlier signs of Aβ accumulation (CSF−/PET− subjects who converted to CSF+/PET− within 2 years), significantly increased Aβ fibril accumulation rate was again seen in the medial orbitofrontal and posterior cingulate cortex, compared with stable CSF−/PET− subjects. These regions were replicated in an independent cohort (*n* = 406). To our knowledge, this is the first study to identify a group of early stage Aβ accumulators and detect increased Aβ accumulation rates specifically in these regions using longitudinal data. Previous longitudinal studies have either failed to detect an early increase in regional Aβ deposition^16^ or identified a rather large early stage Aβ region including the posterior temporal, inferior parietal, posterior cingulate, precuneus, and occipital areas^4^. Those previous results indicate that there might have been methodological issues in correctly identifying the preclinical AD cases who have just started to accumulate Aβ.

There are a few previous studies where cross-sectional results have been used to approximate early stage Aβ accumulation regions. In a subanalysis of a study aimed to identify different Aβ PET cut-offs, voxelwise comparisons were made between a reference group with very low Aβ PET SUVR (*n* = 22) and several groups of subjects (*n* = 22 in each group) with increasingly higher global SUVRs^17^. They found that the first significant SUVR differences between the controls and a group with sub-threshold SUVRs were located in the medial frontal cortex and the precuneus, similar to our early Aβ regions. Another cross-sectional study used a different methodology to approximate the cortical spread of Aβ in 67 healthy controls and 128 patients with MCI or AD dementia^18^. By assuming that earlier regions would show increased Aβ PET signal in a greater number of subjects, they found an initial Aβ region involving the areas in Fig. 1a, but also affecting middle and inferior temporal regions, and superior and middle frontal regions. In neuropathological studies, the results are mixed. One study reported that the presumably first Aβ phase had Aβ deposits throughout the neocortex^5^, while a prior publication by Braak & Braak^19^ showed an early pattern of Aβ deposits in the basal parts of the medial and anterior regions of the prefrontal cortex, and the basal anterior temporal lobe. The differences are probably partly explained by the different staining methods and their affinity to different Aβ forms (discussed in the methodological aspects below). A clear advantage of the present study is that longitudinal Aβ changes in the whole brain has been analyzed, instead of cross-sectional examinations in predetermined regions as in neuropathological studies.

The early Aβ regions we identified were mainly located within the DMN (Fig. 2). The DMN has a core network located in the posterior cingulate cortex, precuneus, and medial prefrontal cortex^20^. It is almost always active, but increasingly so during daydreaming, activation of episodic memory and other internally directed activities and decreasingly during task-based activities and interaction with the external environment^21, 22^. A certain overlap between the DMN and the distribution of Aβ fibrils in AD has also been noted in previous publications^23–26^. The cause of Aβ aggregation within the DMN is currently unknown. One explanation is that the deposition of Aβ fibrils could be caused by the high neuronal activity within the DMN, which is supported by animal studies showing that Aβ secretion and deposition is enhanced by neuronal activity^27–29^. An alternative explanation is that Aβ accumulation is caused by the high metabolic demand and metabolic stress in these neurons (high energy consumption and frequent fluctuations in activation and deactivation) and not the neuronal activation per se^30^. Although the core DMN was the most prominent network that we found that had anatomical overlap with the early Aβ regions, we want to point out that involvement of other networks was also evident in the early Aβ accumulation, especially the frontoparietal network (Fig. 2). This indicates that early stage accumulation is not related to a unique feature of the DMN, but in a more general sense to hubs in the brain with high connectivity. This interpretation is supported by previous studies showing an association between Aβ distribution and several networks and network hubs^25, 31, 32^.

The association between functional networks and earliest stages of Aβ accumulation was further examined using functional connectivity analyses in BioFINDER. When we correlated whole-brain functional connectivity and CSF Aβ42 levels in the early stage Aβ accumulators (CSF+/PET−), we found a decreased connectivity in intra-DMN connections and between DMN and the frontoparietal network as CSF Aβ42 decreased (Fig. 6a). The disruption of these connections in BioFINDER is especially interesting given the distribution of early Aβ fibrils in ADNI in the DMN and frontoparietal networks (Fig. 2). This correlation between the disruption of functional connectivity and Aβ pathology has recently been described in AD^32, 33^, but to our knowledge the present study is the first to show that this association is present already at the very earliest preclinical stages of AD when Aβ fibrils are just starting to accumulate. In our second connectivity analysis (Fig. 6b) we examined the correlation between functional connectivity and CSF Aβ42 in those with indications of very early Aβ accumulation (close to abnormal CSF Aβ42 and normal Aβ PET; CSF-_low_/PET−). The results indicate that when Aβ42 drops towards abnormal levels in the CSF the connectivity instead increases in similar DMN-related connections in which there was a positive correlation between decreased connectivity and decreasing levels of CSF Aβ42 within the abnormal Aβ42 range (compare connectograms in Fig. 4a, b). Interpretations of this preliminary finding are speculative, but it might be that cortical hubs like the medial frontal lobe and posterior cingulate exhibit increased activity before Aβ fibril-containing senile plaques are formed in preclinical AD. This is supported by experimental cell and animal work suggesting that neural activity leads to increased Aβ generation and secretion^27, 34^. Later, the hypoconnectivity might develop when Aβ fibrils starts accumulating and causes neuronal dysfunction. Alternatively, early and soluble Aβ oligomers might instead trigger the initial hyperactivity, which is supported by studies in transgenic AD models suggesting that neurons become hyperactive very early, independently of the deposition of Aβ into plaques, and that the silencing of neurons emerges only later in the disease course^35^.

Although altered functional connectivity was found already in the early Aβ accumulators (CSF+/PET−), our longitudinal analyses (Fig. 4a, c) showed that no changes in glucose metabolism or atrophy were present at this stage. However, decreased temporoparietal glucose metabolism and atrophy was observed in the late Aβ accumulators (CSF+/PET+; Fig. 4b, d and Supplementary Fig. 2A, B). This finding can have practical implications for the enrollment in anti-Aβ clinical trials since anti-Aβ agents should be introduced after Aβ accumulation has started but before neurodegeneration is present. This should make CSF+/PET− subjects suitable for inclusion in clinical trials, especially if the drug is intended to target Aβ accumulation over long time periods using AD biomarkers as outcomes of its efficacy in this preclinical disease stage.

The temporal sequence of Aβ accumulation preceding neurodegeneration confirms the most acknowledged hypothetical model on the development of AD^1^. It also strengthens the rationale for using CSF/PET groups as proxies for Aβ stages in AD. To further validate the use of CSF/PET groups, we compared Aβ accumulation in our proxy group for early Aβ accumulation (CSF+/PET−) with the proxy group for late Aβ accumulation (CSF+/PET+). As hypothesized, we identified known regions of late stage Aβ accumulation, including the sensorimotor cortex and occipital lobe (Fig. 3, Supplementary Movie 2 and Table 3)^3, 19^. In a recent study, we also showed that CSF+/PET− subjects accumulated Aβ at a similar rate as CSF+/PET+ subjects and about four times higher than CSF−/PET− subjects^12^. The high accumulation rate in CSF+/PET− subjects was not accompanied by cognitive decline, but we found a subtle decline in memory over 5 years in CSF+/PET+ subjects that further validates the use of CSF/PET groups for stratifying Aβ stages. Nonetheless, we acknowledge that CSF+/PET− subjects are not all early Aβ accumulators since this state also can be caused by CSF analytical factors, failed PET scans, and medical conditions other than AD^36–38^.

As part of our methodology we chose to primarily compare longitudinal changes between the CSF/PET groups instead of comparing cross-sectional values or just within-group changes to the identify the early Aβ regions. This had the advantage of accounting for physiological differences in Aβ burden, age-related non-specific Aβ accumulation rates, and the fact that the individuals within each CSF/PET stage had reached slightly different time points in the disease progression. In addition, the early Aβ-accumulating brain regions remained significant when adjusting for confounding factors such as longitudinal changes in cortical thickness, *APOE* genotype and clinical status. These adjustments show that the results were not confounded by differences in atrophy between the groups or differences between healthy controls and MCI subjects (discussed further in the Methods section). It should be noted that the early Aβ regions are derived from group analyses, which show where Aβ is prone to start accumulating but they do not exclude the possibility that the accumulation might start in other regions in some individuals.

A limitation of our early Aβ regions is that they have been identified using PET ligands that bind to primarily dense-core Aβ aggregates and to a lesser extent or not at all to diffuse plaques^39, 40^. We can therefore not pinpoint the exact Aβ forms within the early Aβ regions, neither can we say if Aβ aggregates not detected by PET are present in other areas of the brain at this early stage. Future PET ligands that binds to different forms, e.g., Aβ protofibrils, might reveal a different early Aβ pattern^41^. The binding affinity of current PET ligands could explain some of the differences with neuropathological studies that either use silver technique^19^, which stain not only Aβ but also tau, or anti-Aβ antibodies^5^ that bind to specific Aβ epitopes and stain different Aβ forms. Nonetheless, a strength of the methodology is that we could detect similar early Aβ regions in two different cohorts using two different types of PET ligands; ^18^F-florbetapir (a stilbene compound) and ^18^F-flutemetamol (an ^18^F-labeled analog of ^11^C-Pittsburgh Compound B (PiB) derived from thioflavin T)^40^. The cut-offs for the two PET ligands were established separately in each cohort, and the data were not pooled in the analyses, nor were the groups compared to each other. We therefore chose to not transform SUVR into a standardized scale such as centiloid units^42^.

To conclude, we believe that the identified early Aβ regions can have several important implications. These brain regions give a pathophysiological insight into where Aβ fibril accumulation is prone to start in AD and its relationship to functional networks and their connectivity. Our data indicated that Aβ starts accumulating before overt metabolic changes or atrophy, and that hypoconnectivity within the early Aβ-accumulating regions has already occurred when Aβ fibrils just starts to accumulate. The most prominent early Aβ-accumulating regions were the precuneus, posterior cingulate cortex, and the medial orbitofrontal cortex, possibly with an overlap to the anterior cingulate. Based on our results, future studies should be able to examine early unique pathophysiological events and triggering mechanisms of Aβ accumulation by comparing molecular and physiological properties of the early-accumulating regions to later-accumulating regions. These early-accumulating Aβ regions should also be advantageous to use in an Aβ PET composite ROI to better assess early fibrillar Aβ deposition (available at http://biofinder.se), which can have practical implications for early AD diagnostics, Aβ staging and enrollment in clinical trials that target Aβ accumulation.

## Methods

### Study data

Longitudinal data from the ADNI were used for all the statistical analyses in the study, except for replicating the finding of early Aβ accumulating regions and for the functional connectivity analyses for which data from the Swedish BioFINDER study were used. The methodology described in the next sections refers only to analyses performed in the ADNI cohort. Methodology referring to analyses in the BioFINDER cohort is described separately under Methods for the BioFINDER study.

### Participants

Only non-demented individuals were included in this study (characterized as either cognitively normal or diagnosed with mild cognitive impairment, MCI). Demented subjects were excluded since the study aim was to identify early AD pathology. We chose to include MCI subjects in addition to healthy controls since the cognitive impairment did not necessarily have to be caused by an underlying AD process and because about a third of MCI subjects have shown to be incorrectly diagnosed with cognitive impairment^43–45^. We also attempted to include a range of different pre-dementia AD phases for a better staging of late Aβ accumulators, early Aβ accumulators and non-accumulators. To account for this inclusion the models were adjusted for clinical status (MCI/healthy control) in subanalyses. The specific inclusion/exclusion criteria for the ADNI cohort can be found at http://www.adni-info.org. Briefly, all subjects were enrolled from the ADNI-2 study, were between the ages of 55 and 90 years, were fluent in Spanish or English, had completed at least six years of education, had a Mini-Mental State Examination score (MMSE) of ≥24^46^, and were free of any significant neurologic disease other than AD. Subjects classified as cognitively normal (*n* = 176) had a Clinical Dementia Rating scale (CDR) score of 0^47^. MCI (*n* = 297) was defined as having preserved activities of daily living, absence of dementia, and an objective cognitive impairment as shown on the delayed recall test of the Wechsler Memory Scale - Logical Memory II as well as a CDR score of 0.5. Only subjects with a complete set of baseline and follow-up Aβ PET scans and baseline CSF Aβ42 data were included. Our study baseline was defined as the first visit where both CSF and PET data were available. Study subjects gave written informed consent and the study was approved by each participating site’s Institutional Review Board.

### Image acquisition

For MRI, 3 Tesla scanners were used. High-resolution 3D T1-weighted images were acquired for volumetric measures, anatomical segmentation, and template normalization using an MPRAGE sequence (for details, see ref. ^48^). Fibrillar Aβ pathology was measured using the PET ligand ^18^F-florbetapir. Image data were acquired 50–70 minutes post-injection (see http://adni.loni.usc.edu/wp-content/uploads/2010/05/ADNI2_PET_Tech_Manual_0142011.pdf). Glucose metabolism was measured employing FDG PET with images acquired 30–60 min post-injection. Florbetapir PET, FDG PET, and MR images from baseline and the 2-year follow-up visit were downloaded from the LONI database (http://ida.loni.usc.edu) and further processed locally. Longitudinal ^18^F-florbetapir was available in all subjects, longitudinal MRI in 468 subjects (218 CSF−/PET−, 59 CSF+/PET−, and 191 CSF+/PET+), and longitudinal FDG PET in 318 subjects (153 CSF−/PET−, 41 CSF+/PET−, and 124 CSF+/PET+).

### Image processing

The motion corrected PET data was time-averaged and rigidly aligned with the anatomical MRI scan. SUVR were then calculated by normalizing to averages of appropriate reference regions. For florbetapir, the reference region consisted of the whole cerebellum, the pons/brainstem region, and eroded cortical white matter as this composite region has produced more reliable results in longitudinal analyses^49^. For FDG, a composite region comprising vermis/cerebellum superior to the horizontal fissure and pons were used as reference region^50^. Freesurfer version 5.3 (http://surfer.nmr.mgh.harvard.edu; Desikan-Killiany Atlas) segmentations of the structural MR images were applied to extract SUVRs from 68 predefined ROIs. Partial volume error (PVE) correction was performed for florbetapir SUVRs with the geometric transfer matrix (GTM) method^51^ using Freesurfer segmented regions^52^ (all florbetapir results shown in the tables and figures are from PVE corrected data).

The MRI volumes were further normalized to MNI152 standard space^53^ using a nonlinear diffeomorphic transformation^54^ enabling motion and PVE voxelwise^55^ corrected SUVRs to be transformed to MNI standard space for voxelwise analyses. VBM as implemented in Statistical Parametric Mapping (SPM) version 12 (Wellcome Department of Cognitive Neurology, London, UK; http://www.fil.ion.ucl.ac.uk/spm) was used to evaluate GM intensity as a measure of atrophy. All individual T1-weighted MR images were segmented into tissue classes using SPM’s unified segmentation algorithm. The GM segmentations were subsequently warped into a common MNI152 standard space using a cohort-specific template and the SPM Diffeomorphic Anatomical Registration Through Exponentiated Lie Algebra (DARTEL) toolbox and Jacobian scaled to estimate GM intensity. The resulting maps were smoothed with 8 mm full-width at half maximum (FWHM) Gaussian kernel. Intracranial volume (ICV) was calculated by summating the volumes of GM, white matter, and CSF based on the SPM tissue segmentations. Using the corrected SUVRs in standardized space, we also proceeded to calculate summed values within the functional MRI (fMRI) resting-state networks enumerated in the low resolution atlases with seven networks calculated by Yeo et al. (see details below)^15^.

Florbetapir and FDG PET images were co-registered to their respective MRI and subsequently warped into a common MNI152 space using the transformation information derived from warping the MRI scans. To assess changes over time in GM intensity (MRI), amyloid accumulation (florbetapir PET), and glucose metabolism (FDG PET) between baseline and follow-up examinations, parametric change maps were created. For longitudinal VBM, a midpoint average MRI was created for each subject based on the baseline and follow-up images using SPM12, resulting in Jacobian determinant maps showing local volumetric change between baseline and follow-up for each subject. Midpoint average images were GM-segmented and subsequently used to create a DARTEL template that was affine-transformed to the common MNI152 space. Midpoint average GM segmentations were multiplied by the Jacobian maps to create individual GM change maps, which were warped to the MNI-transformed DARTEL template and smoothed by an 8 mm FWHM isotropic Gaussian kernel to create longitudinal change maps. A GM mask was created using the same approach as for the cross-sectional data (see above). The florbetapir and FDG change maps were created using the warped images and FSL (http://fsl.fmrib.ox.ac.uk) according to the equation

$${\rm{SUV}}{{\rm{R}}_{{\rm{change}}}} = {\rm{(PET}}\,{\rm{imag}}{{\rm{e}}_{{\rm{followup}}}} - {\rm{PET imag}}{{\rm{e}}_{{\rm{baseline}}}}{\rm{)/years}}\,{\rm{between}}\,{\rm{scans}}$$

### CSF biomarkers

CSF Aβ42, total tau (T-tau), and tau phosphorylated at Thr181 (P-tau) were measured using the multiplex xMAP Luminex platform (Luminex Corp, Austin, TX, USA) with the INNOBIA AlzBio3 kit (Innogenetics, Ghent, Belgium)^56, 57^. Data was combined from the available online files “UPENNBIOMK8.csv”, “UPENNBIOMK7.csv, “UPENNBIOMK6_07_02_13.csv”, and “UPENNBIOMK5_10_31_13.csv”. Only cross-sectional CSF data from baseline were used in the analyses (*n* = 473), except for the analysis of “CSF converters” where longitudinal 2-year data was used (*n* = 112). In the case of one subject with stable Aβ42 levels, only 3-year follow-up data was available. The mean difference between baseline CSF sampling and the florbetapir PET scan was 9.6 days (ranging from 0 to 110 days).

### Group classifications

All subjects were categorized into different groups according to their CSF Aβ42 and Aβ PET status. The following groups were derived from the baseline results: (1) normal CSF Aβ42 and Aβ PET (CSF−/PET−) referred to as non-accumulators (*n* = 218), (2) abnormal CSF Aβ42 and normal Aβ PET (CSF+/PET−) referred to as early Aβ accumulators (*n* = 59), and (3) abnormal CSF Aβ42 and Aβ PET (CSF+/PET+) referred to as late Aβ accumulators (*n* = 191). Those with normal CSF Aβ42 and abnormal Aβ PET were not used in any analysis (*n* = 5). Abnormal CSF Aβ42 values were defined using the previously established cut-off of <192 ng/L^57–59^. The cut-off for abnormal Aβ PET SUVR was determined using a mixture modeling analysis since no previous cut-off had been established for PVE corrected florbetapir data in ADNI (using the GTM method). Mixture modeling statistics provide an unbiased threshold for abnormality (i.e., the diagnosis or status is not used in analysis) and it is a well-established method for defining Aβ PET cut-offs^12, 17, 60, 61^. To achieve a robust threshold we derived the cut-off from a larger ADNI sample (*n* = 663, all non-demented subjects with CSF and Aβ PET data). This resulted in a cut-off of >0.872 SUVR for an abnormal Aβ PET using PVE correction and the composite reference region.

### Methods for the BioFINDER study

The Swedish BioFINDER study was used for replicating the main results from ADNI and for the functional connectivity analyses. BioFINDER is a prospective study that focuses on identifying key mechanisms and improvement of diagnostics in AD and other neurodegenerative disorders. For details about study design, methods, and specific inclusion/exclusion criteria, see http://biofinder.se. The study was approved by the ethical review board in Lund, Sweden, and all participants gave their written informed consent. All non-demented individuals with CSF and Aβ PET data were selected for this study. This resulted in a cohort consisting of cognitively healthy elderly subjects (*n* = 138) and consecutively recruited patients who had been referred to memory clinics due to cognitive complaints (*n* = 268). The PET ligand ^18^F-flutemetamol was used for Aβ PET, and images were acquired 90–110 min post-injection. The PET scanning procedures have been described previously^60^. CSF Aβ42 and P-tau were analyzed with INNOTEST ELISAs (Fujirebio Europe, Ghent, Belgium) and T-tau with EUROIMMUN ELISAs (EUROIMMUN AG, Lübeck, Germany) as previously described^60, 62^. Mixture modeling was performed in the sample to determine the cut-offs for abnormal CSF Aβ42 (<517 ng/L) and abnormal Aβ PET (>0.759 SUVR). Only baseline data was available for analysis in the BioFINDER cohort.

Imaging was performed on a 3 Tesla Siemens Tim Trio scanner (Siemens Medical Solutions, Erlangen, Germany). The high-resolution 3D T1-weighted volume used for segmentation and normalization was acquired using an MPRAGE sequence (in-plane resolution = 1 × 1 mm^2^, slice thickness = 1.2 mm, TR = 1950 ms, TE = 3.37 ms, flip-angle = 9°). Spontaneous BOLD oscillations in the absence of external stimuli were imaged with a gradient-echo planar sequence (eyes closed, in-plane resolution = 3 × 3 mm^2^, slice thickness = 3mm, TR = 2000 ms, TE = 30 ms, flip-angle = 90°, 180 dynamic scans, 6 min).

Resting-state data preprocessing was performed with a pipeline composed of FSL^63^, AFNI^64^, and ANTS^65^. Anatomical processing involved skull stripping, segmentation of white matter (WM)/GM/CSF and normalization to MNI152-space^53^. Dropping the first five frames in anticipation of steady state, functional data was bulk motion, and slice timing corrected, furthermore nuisance regressed using the WM/CSF average signal, 6 components of physiological noise^66^, 24 motion parameters^67^, and linear/quadratic trends. Finally, the functional data was transformed to MNI space. Frames causing outliers in total frame-to-frame signal variation were censored, on average constituting 4% of the fMRI series^68^. The signal was band-pass filtered to 0.01–0.1 Hz, further discriminating against scanner drift and physiological noise. No spatial smoothing was applied.

Subjects with a mean/maximum frame-wise displacement^29, 69^ exceeding 0.6/3.0 mm were excluded. As an extra precautionary step, the voxel-to-voxel BOLD-signal correlations across the whole brain (including GM, WM, and CSF) was calculated and summed. Outliers in this measure likely originate in a motion-induced global signal confound capable of eluding conventional motion estimation^70^ and were removed.

The processed fMRI data was resampled using trilinear interpolation to 6 × 6 × 6 mm^3^ resolution and masked with GM derived from a cortical resting-state network atlas^71^ and Harvard-Oxford subcortical atlas^72^. Fisher-z transformed Pearson correlation between the resulting 5071 GM voxel time series then yielded a measure of functional connectivity (FC), corresponding to a weighted graph with nodes (voxels) and links (voxel BOLD time series correlations).

Network components correlating with CSF Aβ42 were calculated using a method similar to the NBS algorithm^73^. We calculated the largest network component *C* (defined as a connected set of links), for which *r*_*ij*_ > *r*_0_, where *r*_*ij*_ is the Spearman correlation over all subjects between CSF Aβ42 and *z*_*ij*_ for the link between voxel *i* and *j* (Fisher z-transformed voxel BOLD time-series correlation), and *r*_0_ controls the component size and significance level of constituent links. We chose *r*_0_ corresponding to approximately *p* = 0.001 given the number of subjects in the calculation. Component size was then defined as the sum of all ${r_{ij}}{\it{\epsilon }}C$ multiplied by the Spearman correlation of the sum of all *z*-values in the component and CSF levels. This last step allows for reasonable and natural variations on the extracted component. The component size was compared to a permutation-generated null distribution of sizes, thus controlling for the family-wise error rate in the weak sense at *α* = 0.05 The result of the algorithm is a network component on which sum of *z*-scores correlates significantly higher than for randomized sets of subject FC-CSF pairs. Age, gender and *APOE* ε4 status was controlled for by partial correlation.

In order to simplify the analysis of network components, we grouped nodes using a resting-state network atlas^71^ containing: default mode, dorsal and ventral attention, sensory motor, visual, fronto-parietal, and fronto-temporal (medial temporal lobe/orbitofrontal cortex). To this set of labels we added two anatomically defined subcortical structures from the Harvard-Oxford atlas^72^: the Basal Ganglia (BG: thalamus, caudate, putamen and pallidum) and hippocampus/amygdala (HI). Note that the permutation-based approach generates *p* values for the network component as a whole, but since these are too large and complex to visualize, a network-based break up is necessary.

Only Aβ PET negative subjects were used in the connectivity analysis. In addition to the previously described group of early Aβ accumulators (CSF+/PET−, *n* = 23 after fMRI quality control) we also defined a group of biomarker negative subjects with indications of very early Aβ accumulation (*n* = 80). Those with low levels of CSF Aβ42, but still within the normal range, have a high risk of becoming abnormal within the next couple of years^13^, which suggests that sub-threshold CSF Aβ42 levels indicate very early Aβ accumulation. This group was characterized as Aβ PET negative with CSF Aβ42 between 517–750 μg/mL (CSF−_low_/PET−).

### Statistical analysis

Group comparisons of baseline characteristics (Table 1 and Supplementary Table 2) was performed with Mann–Whitney *U* statistics if significant after the Kruskal–Wallis test. For ROI analyses, full factorial general linear models were used to analyze group differences and correlations of the 68 available cortical Freesurfer ROIs from the Desikan-Killiany Atlas. The dependent variable was Aβ accumulation rate ([SUVR at follow-up – SUVR at baseline]/years between baseline and follow-up) in ADNI and baseline Aβ PET SUVR in BioFINDER. The variance of the SUVRs were similar in each group. All results were adjusted for gender and age and, in the longitudinal analyses, time between Aβ PET examinations. We also performed additional analyses adjusting for changes in cortical thickness over 2 years in each ROI and clinical status (healthy control/MCI) in addition to the above covariates. Longitudinal changes within a specific group of subjects were analyzed with the Paired T-test. Because of the limited number of subjects in the subanalysis of “CSF converters” (*n* = 11), we primarily used non-parametric statistics for this subgroup (the Mann–Whitney *U* and Wilcoxon signed rank test). To control for multiple testing in the ROI-based analyses we used the Benjamini–Hochberg procedure with a conservative false discovery rate (FDR) of 0.05 (not to be confused with a *p* value of 0.05)^74^. Original *p* values are presented for the ROI-based analyses but are only reported as significant if they are significant after the FDR correction. The effect size of the significant results when comparing the CSF/PET groups are given in % annual increase of SUVR (yearly SUVR change divided by SUVR at baseline) for an easier comparison between different PET ligands and brain regions (and when comparing the results to other studies that use different reference regions). However, statistical significances were determined using actual SUVR or SUVR/year data. SPM12 was used for all voxelwise analyses. We employed voxelwise two-sample *t*-test and multiple regressions using age, gender, and ICV (for analyses involving VBM data) as covariates. All images had been transformed into common MNI space by using transformation measures from warping the co-registered MRI scans to the 2 mm MNI152 MRI template and smoothed with an 8 mm FWHM kernel. The resulting t-maps were thresholded to present results with a *p* < 0.001 height, uncorrected for family-wise error, and a *k* > 100 voxels cluster extent. For the resting-state correlation analysis, a component (consisting a connected set of links correlating with CSF biomarker levels above a *p* = 0.001 threshold) was assigned a *p* value as a whole based on the component size relative a permutation-generated null distribution, thus correcting for family-wise cerror in the weak sense (see “Methods for the BioFINDER study” for details). Finite mixture models for establishing biomarker cut-offs were performed with the package “mixtools” in R version 3.2.2 (R Foundation for Statistical Computing, Vienna, Austria, 2013). FDR correction of multiple comparisons was performed using the Benjamini–Hochberg formula in an Excel sheet (Microsoft Excel for Mac, 2011, version 14.4). All other statistical analyses were performed with SPSS for Mac, version 22.0 (SPSS Inc., Chicago, IL).

### Data availability

MRI and PET images were downloaded online at https://ida.loni.usc.edu and further processed locally (see Image Processing above). All other ADNI data were also downloaded from the same site. BioFINDER data and processed ADNI data are not publicly available for download, but might be retrieved from the principal investigator Oskar Hansson. A PET-data-derived ROI of the early-accumulating Aβ regions (Fig. 1a) is available in MNI152 space and Nifti-format at http://biofinder.se.

## Electronic supplementary material

Supplementary information

Description of Additional Supplementary Files

Supplementary Movie 1

Supplementary Movie 2

Supplementary Movie 3
