# Supplementary material for: Earliest accumulation of β-amyloid occurs within the default-mode network and concurrently affects brain connectivity
Source: Nat Commun. 2017 Oct 31;8:1214. doi: 10.1038/s41467-017-01150-x (PMC5663717; doi:10.1038/s41467-017-01150-x)
Supplement: Supplementary file 1 — Supplementary information [file 41467_2017_1150_MOESM1_ESM.pdf]

Supplementary tables

Supplementary Table 1. Early Aβ regions when adjusting for APOE ε4 allele

**Brain Region**	***p* value**
Posterior cingulate cortex, right	0.000051
Isthmus cingulate cortex, left	0.00026
Isthmus cingulate cortex, left	0.00038
Isthmus cingulate cortex, right	0.00051
Medial orbitofrontal cortex, left	0.00081
Rostral anterior cingulate cortex, right	0.0011
Transverse temporal gyrus, right	0.0025
Posterior cingulate cortex, left	0.0027
Medial orbitofrontal cortex, right	0.0031
Insula, right	0.0032
Lateral orbitofrontal cortex, left	0.0041
Precuneus, right	0.0053
Rostral anterior cingulate cortex, left	0.0074
Superior frontal cortex, left	0.0085
Insula, left	0.010

Comparisons in ADNI between early Aβ accumulators (CSF+/PET-) and non-accumulators (CSF-/PET-) subjects using general linear models with the Aβ PET SUVR change/year. The analysis was the same as in Table 2 (main article), but here we also adjusted for presence of APOE ε4 allele (one or two) in addition sex, age and time between PET.
Abbreviations: CSF+, Cerebrospinal fluid Aβ <192 ng/L; PET+, >0.87 SUVR; SUVR, standardized uptake value ratio (using a composite reference region).

Supplementary Table 2. Characteristics of the BioFINDER cohort

	**Entire BioFINDER sample**	**(A) CSF−/PET−**	**(B) CSF+/PET−**	**(C) CSF+/PET+**	***p* value**
N	406	219	30	135	
Baseline CN/SCD/MCI (%)	34/29/36	46/32/22	43/20/37	15/29/56	A-B=0.35<br>**B-C=0.005**<br>**A-C<0.001**
Age (years)	71.5 (5.4)	71.0 (5.6)	71.7 (5.9)	71.9 (5.0)	0.38
Gender (women)	49%	53%	50%	46%	0.44
Education (yrs)	11.8 (3.4)	12.0 (3.3)	11.4 (4.0)	11.6 (3.3)	0.21
Presence of *APOE ε4* allele	40%	21%	34%	73%	A-B=0.11<br>**B-C<0.001**<br>**A-C<0.001**
MMSE (0–30 points)	28.2 (1.6)	28.6 (1.5)	28.2 (1.6)	27.7 (1.7)	A-B=0.31<br>B-C=0.09
10-word delayed recall (0–10 errors)	4.0 (2.8)	3.0 (2.4)	3.8 (2.5)	5.4 (2.8)	A-B=0.06<br>**B-C=0.003**<br>**A-C<0.001**
Neocortical composite flutemetamol SUVR[1]	0.81 (0.29)	0.61 (0.06)	0.64 (0.10)	1.14 (0.22)	**A-B=0.004**<br>**B-C<0.001**<br>**A-C<0.001**
Hippocampus volume, cm^3	7.1 (1.0)	7.3 (0.9)	7.1 (0.7)	6.8 (1.0)	A-B=0.12<br>B-C=0.23
CSF Aβ42 (ng/L)	606 (219)	770 (135)	414 (85)	378 (77)	**A-B<0.001**<br>**B-C=0.01**<br>**A-C<0.001**
CSF T-tau (ng/L)	364 (165)	286 (77)	318 (147)	492 (190)	A-B=0.89<br>**B-C<0.001**<br>**A-C<0.001**
CSF P-tau (ng/L)	59 (26)	49 (14)	52 (26)	77 (31)	A-B=0.94<br>**B-C<0.001**<br>**A-C<0.001**

Values are given in mean (SD) if not otherwise specified. Specific group comparisons were made with Mann-Whitney U statistics if significant after the Kruskal-Wallis test. Significant p values are in bold. The CSF-/PET+ subjects were not included in the table as a separate group since they were not included in any analysis.
CN, cognitively normal; CSF, cerebrospinal fluid; MCI, mild cognitive impairment; MMSE, Mini Mental State Examination; N, number of subjects; SCD, subjective cognitive decline; SD, standard deviation

Supplementary Table 3. Aβ SUVRs of the significant early Aβ regions in BioFINDER

**Region**	**CSF+/PET-**	**CSF-/PET-**	***p* value**
Medial orbitofrontal cortex, left	0.60 (0.11)	0.52 (0.08)	0.00000075
Posterior cingulate cortex, right	0.68 (0.15)	0.61 (0.09)	0.00013
Rostral middle frontal cortex, right	0.72 (0.14)	0.65 (0.09)	0.00014
Precuneus, left	0.61 (0.14)	0.55 (0.09)	0.00017
Lateral orbitofrontal cortex, left	0.66 (0.11)	0.60 (0.08)	0.00021
Medial orbitofrontal cortex, right	0.61 (0.13)	0.54 (0.09)	0.00027
Rostral middle frontal cortex, right	0.72 (0.14)	0.65 (0.09)	0.00028
Posterior cingulate cortex, left	0.68 (0.10)	0.62 (0.08)	0.00058
Frontal pole, right	0.81 (0.21)	0.71 (0.16)	0.00058
Precuneus, right	0.59 (0.17)	0.53 (0.09)	0.0020
Pars orbitalis, left	0.69 (0.12)	0.63 (0.09)	0.0028
Inferior parietal lobe, right	0.67 (0.15)	0.62 (0.08)	0.0028
Isthmus cingulate cortex, right	0.69 (0.18)	0.63 (0.09)	0.0067
Inferior temporal lobe, right	0.66 (0.10)	0.62 (0.07)	0.0091
Inferior parietal lobe, left	0.70 (0.14)	0.65 (0.08)	0.010
Rostral anterior cingulate cortex, left	0.63 (0.13)	0.58 (0.09)	0.010
Inferior temporal lobe, left	0.67 (0.07)	0.63 (0.07)	0.011
Pars orbitalis, right	0.66 (0.11)	0.62 (0.09)	0.013

Comparison between CSF+/PET- and CSF-/PET- subjects in general linear models with ^{18}F-flutemetamol SUVR as the dependent variable and CSF/PET groups, sex and age as co-variates. Data are given in mean values (95% CI of mean) in order of significance. Only significant regions after the Benjamini & Hochberg correction are shown.

Supplementary Figures

Supplementary Figure 1. Whole-brain axial images of the significant early Aβ regions in ADNI

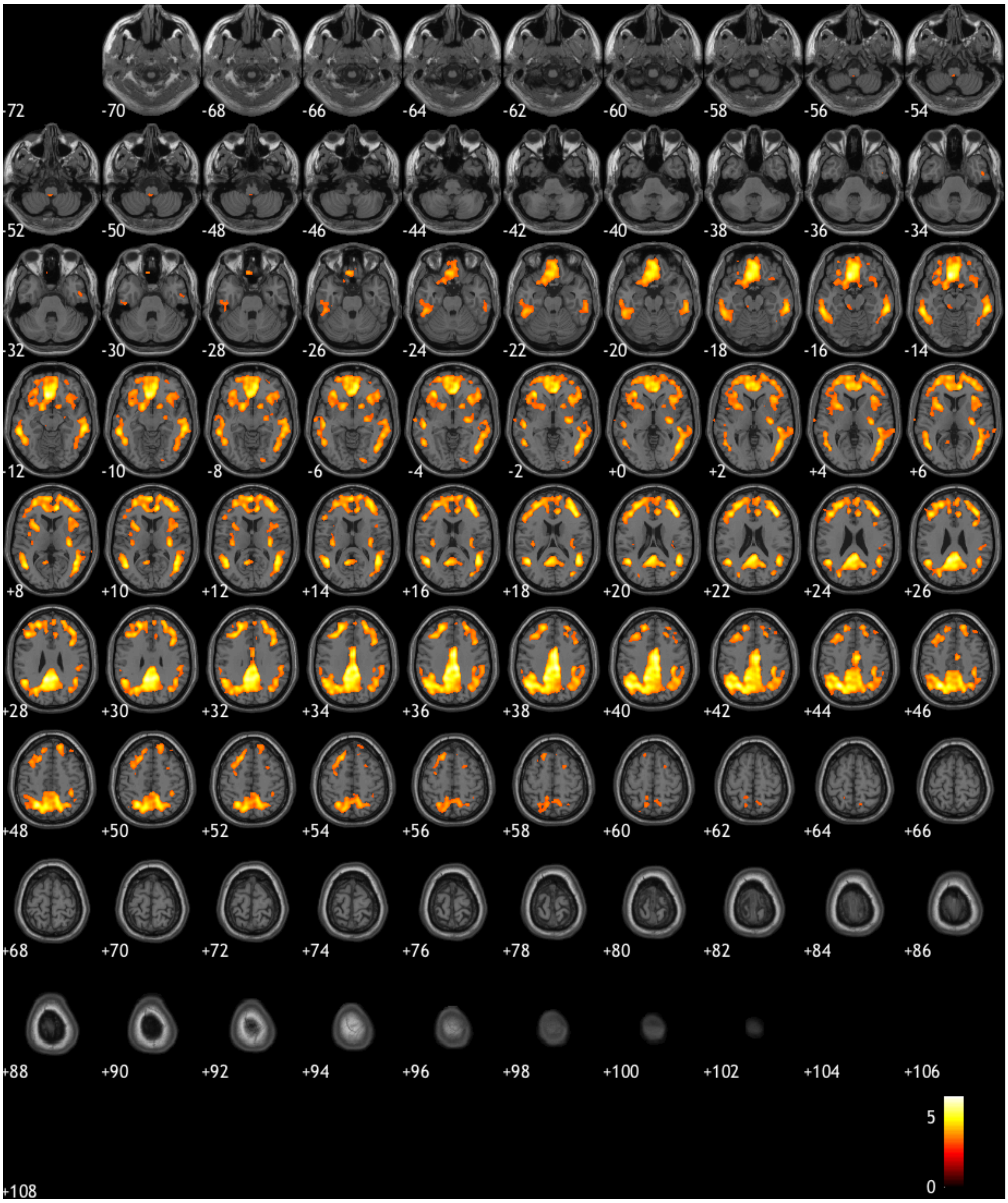

Voxelwise comparison of annual florbetapir SUVR rate between early accumulators (CSF+/PET-) and non-accumulators (CSF-/PET-) to identify early Aβ regions (as in Fig. 1A). The significant threshold was set at p<0.001 and the comparison was adjusted for age and gender. The colors depict the significant t values.

Supplementary Figure 2. Longitudinal atrophy and metabolism comparisons between early and late Aβ accumulators in ADNI

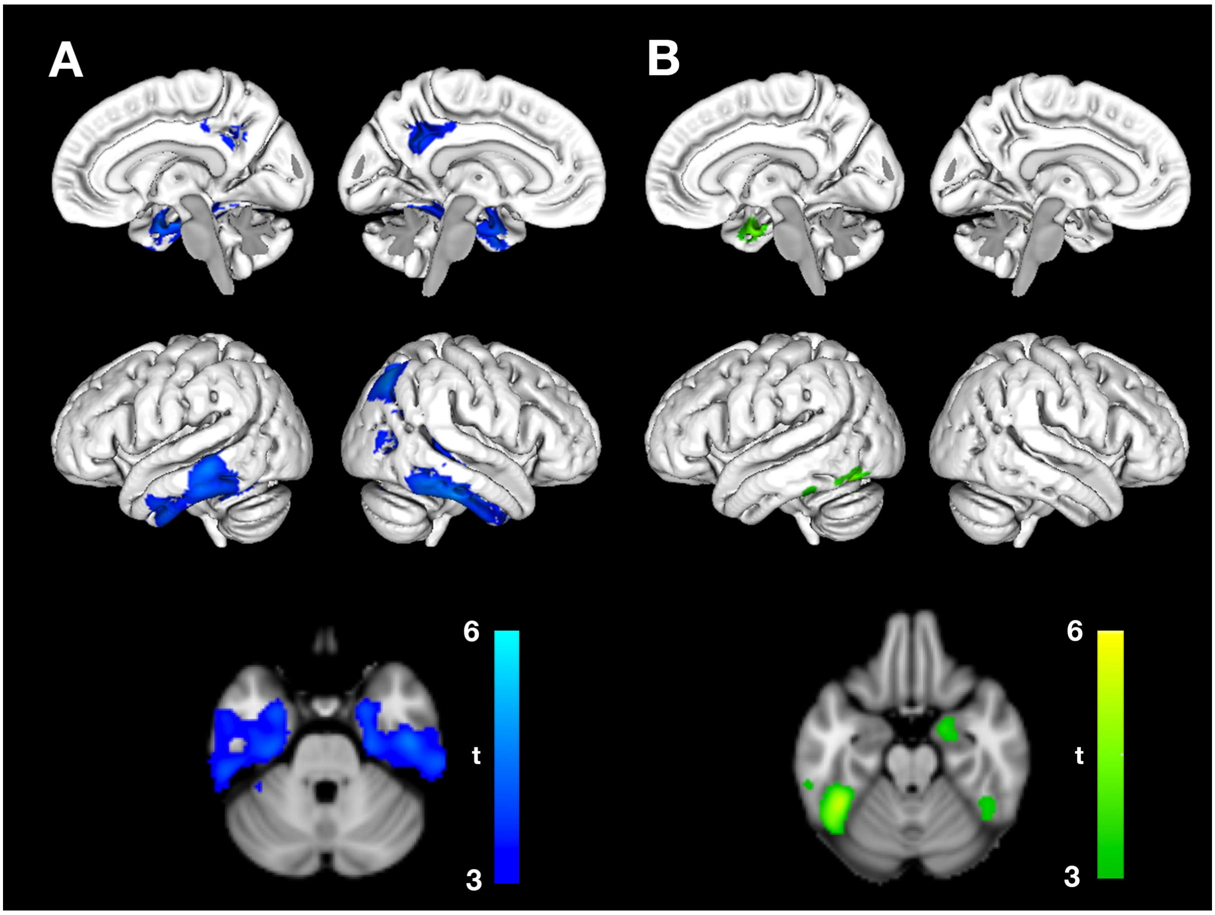

A. *Voxel-based morphometry analysis of annual change in cortical volumes showing atrophy in temporal and to a lesser extent parietal regions in late Aβ accumulators compared with early Aβ accumulators.* ***B***. *Voxelwise comparison of annual FDG PET change showing reduced glucose metabolism in the temporal lobes. All comparisons were adjusted for age and gender and the significance threshold was set at p<0.001. The colors illustrate significant t values according to the scales.*

Supplementary Figure 3. Functional connectivity association in BioFINDER adjusted for tau

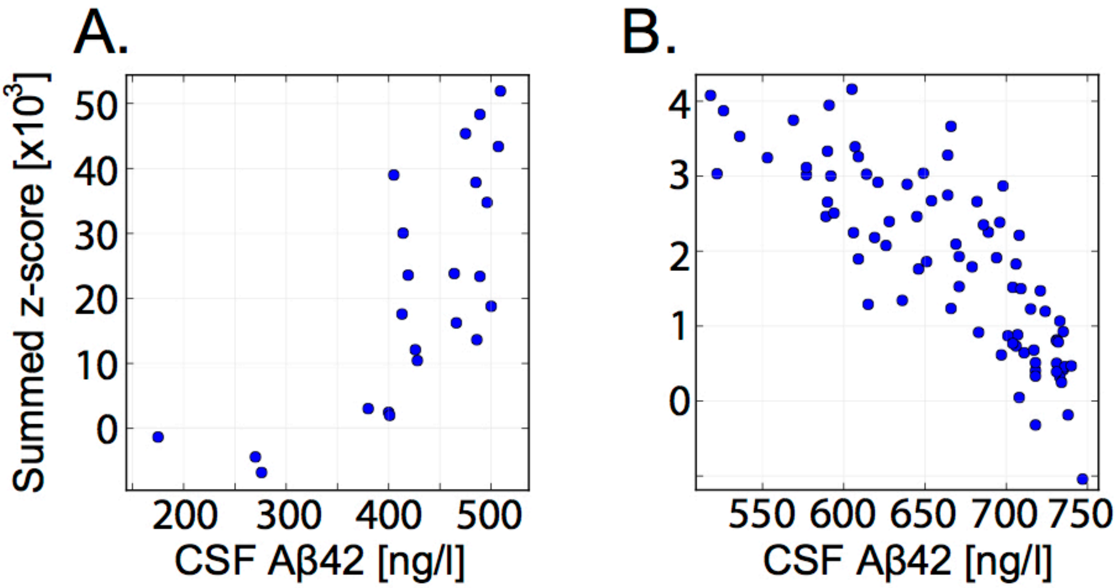

CSF Aβ42 correlations with whole brain resting-state fMRI connectivity in non-demented BioFINDER subjects with normal Aβ PET and QC-passed fMRI data (n=103). In addition to the analysis in Fig. 6 (main article), these correlations have also been adjusted for CSF P-tau to show the independent association between early Aβ accumulation and connectivity. ***A*** *shows the correlation between reduced connectivity and decreasing CSF Aβ42 levels in the CSF+/PET- group.* ***B*** *shows the correlation between increased connectivity and decreasing CSF Aβ42 levels in the CSF-$_{low}$/PET- group.*

Ackronym: CSF-$_{low}$, normal CSF Aβ42 levels close to the abnormal cutoff (517–750 ng/L)
