# Supplementary material for: Earliest accumulation of β-amyloid occurs within the default-mode network and concurrently affects brain connectivity
Source: Nat Commun. 2017 Oct 31;8:1214. doi: 10.1038/s41467-017-01150-x (PMC5663717; doi:10.1038/s41467-017-01150-x)
Supplement: Supplementary file 2 — Description of Additional Supplementary Files [file 41467_2017_1150_MOESM2_ESM.pdf]

Description of Additional Supplementary Files

File Name: Supplementary Movie 1
Description: Illustration of the early Aβ accumulation regions from the ROI-based analysis in ADNI (Table 2). Comparisons were made between CSF+/PET- and CSF-/PET- subjects using general linear models with the Aβ PET SUVR change/year as the dependent variable and CSF/PET groups, sex, age and time between PET scans as co-variates. Only the 15 significant regions after the Benjamini & Hochberg correction are shown.

File Name: Supplementary Movie 2
Description: Illustration of late Aβ accumulation regions from the ROI-based analysis in ADNI (Table 3). Comparisons were made between CSF+/PET- and CSF+/PET+ subjects using general linear models with the Aβ PET SUVR change/year as the dependent variable and CSF/PET groups, sex, age and time between PET scans as co-variates. Only significant regions after the Benjamini & Hochberg correction are shown.

File Name: Supplementary Movie 3
Description: Illustration of the early Aβ accumulation regions from the ROI-based analysis in BioFINDER (Supplementary Table 2). Comparisons were made between CSF+/PET- and CSF-/PET- subjects using general linear models with Aβ PET SUVR as the dependent variable and CSF/PET groups, sex and age as co-variates.
